# Revolutionizing Cardiovascular Interventions With Artificial Intelligence

**DOI:** 10.1016/j.jscai.2025.102580

**Published:** 2025-03-18

**Authors:** Yiannis S. Chatzizisis, Elazer R. Edelman

**Affiliations:** a Center for Digital Cardiovascular Innovations, Division of Cardiovascular Medicine, Miller School of Medicine, University of Miami, Miami, Florida; b Institute for Medical Engineering and Science, Massachusetts Institute of Technology, Cambridge, Massachusetts

**Keywords:** artificial intelligence, cardiovascular interventions, digital twins, education, preprocedural planning

The field of cardiovascular interventions has witnessed remarkable advancements over the past few decades, with revolutionary breakthroughs in vascular biology and pharmacology on the one hand and in materials science and interventional medicine on the other. As we stand on the cusp of a new era, computational science has advanced from primitive machine learning to advanced and rapidly evolving artificial intelligence (AI). With these advancements comes the promise to redefine health care along another set of revolutionary lines.

As in all aspects of life and perhaps to an even greater degree in medicine, AI eases and contributes to current treatment paradigms and offers the opportunity to address long-standing challenges and open new avenues for previously inconceivable ideas. AI is set to become a driving factor in the evolution of interventional cardiology, as it can help optimize procedural planning, accelerate device development, and enhance disease characterization, diagnosis, and education, while breaking disparities in access (Figure 1). This issue of *JSCAI* presents a series of articles that touch on these elements and show how AI is changing and will change the practice of interventional cardiology.

**Figure 1:**
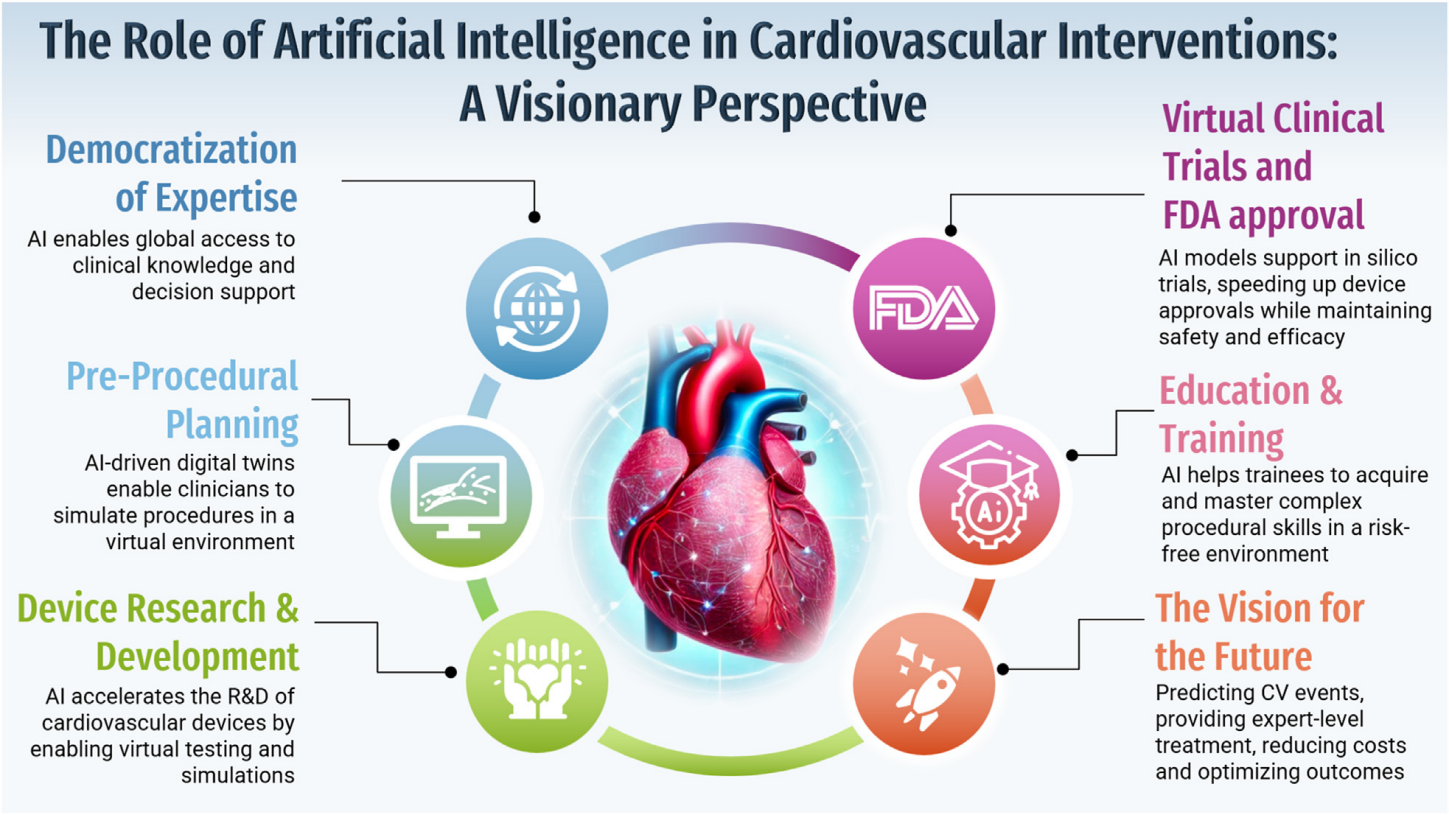
**Role of AI in cardiovascular interventions.** AI, artificial intelligence; CV, cardiovascular.

## Democratization of expertise and knowledge

Perhaps, the greatest boon of AI is its universal availability and the potential it has to bridge the knowledge and skills gap in interventional cardiology by democratizing access to clinical expertise. Historically, advanced procedural skills and insights have been concentrated in specialized centers and in the hands of a limited number of experts—adhering to ancient rituals of passing advances directly from mentor to student. Not only transmission and preservation of ideas and innovations were then limited, but there was also a risk of loss and dissipation. AI-driven systems can break down these barriers, disseminating knowledge to a global audience and fostering equitable access to high-quality care, enabling almost instantaneous enhancement and amplification. As noted in the articles by Alsharqi and Edelman^1^ and by Petraco et al,^2^ the integration of AI into imaging modalities can assist with preprocedural planning, enabling less experienced operators to perform sophisticated procedures with greater confidence and precision.^3^ Similarly, Shin et al^4^ and Young et al^5^ explain how AI-powered clinical decision support systems can integrate the unimaginable mass of knowledge from basic biology to current pathophysiologic insights can drive real-time image analysis, segmentation, and annotation to guide operators in selecting the most appropriate treatment strategies for individual patients. With equal ease, AI can factor in variables from anatomical complexity, comorbidities, and procedural risks and every imaginable metric and parameter, thereby reducing interoperator variability and improving clinical outcomes. The time will soon arrive when AI drives semiautomated and even fully autonomous procedures, for now George et al^6^ detail how robot-assisted interventions appear to be directly facilitated by AI-powered procedural planning.

## Preprocedural planning using digital twins

Computational approaches like AI rely on data, and confidence from such systems are powered by data access and limited by data paucity. Here then is another advantage of AI—the ability to expand data sets from limited density cohorts. The pieces by Samant et al,^7^ Elias et al,^8^ and Aminorroaya et al^9^ add to a growing literature that explains how digital twinning, the creation of virtual replicas of a physical entity, are being extended to population science to enlarge the data sets available for analysis in interventional cardiology from small samples of images and patients.^3^^,^^10^ This approach is vital to interventional cardiology. By integrating even limited amounts of patient-specific data, including imaging, hemodynamic parameters, and genomic profiles, AI-driven digital twins enable operators to simulate procedures in a virtual environment. When coupled with virtual interventions, digital twinning can be used to predict potential complications and outcomes, empowering operators to refine their approach in advance. This not only improves procedural efficiency and precision but also personalizes care and has the potential to lower overall health care costs while improving clinical outcomes. For example, in percutaneous coronary interventions, patient-specific in silico models can assist in endovascular planning, from lesion preparation to stent sizing and positioning, minimizing adverse events, such as restenosis or thrombosis.^3^^,^^11^ Similarly, in structural heart interventions, such as transcatheter aortic valve replacement, digital twins can simulate valve deployment, predict paravalvular leak, and optimize device sizing.^3^^,^^12^

## Shortening cardiovascular device lifecycle

Artificial intelligence is also poised to revolutionize the total product lifecycle of cardiovascular devices, from research and development to regulatory approval and postmarket surveillance.^3^

Developing innovative cardiovascular devices traditionally involves extensive bench testing, animal studies, and human trials, often spanning years. AI can accelerate this process by enabling in silico testing, where virtual models replicate the physical and biological behavior of devices under various conditions.^13^ This approach allows researchers to evaluate device performance, identify potential flaws, and refine designs without the need for costly and time-consuming physical prototypes. For example, stent designs can be tested for durability and hemodynamic performance in virtual environments, accelerating the time from inception to clinical use.

Regulatory bodies, like the US Food and Drug Administration, are increasingly recognizing the value of virtual (in silico) clinical trials as a supplement (or even alternative) to traditional clinical trials.^3^^,^^14^ AI models can simulate patient populations, predict device outcomes, and generate robust data sets to support regulatory submissions, enabling the acquisition of new knowledge in a time-effective and cost-effective manner. By reducing the dependency on extensive clinical trials, virtual clinical trials can shorten the time to market for life-saving devices while maintaining rigorous safety and efficacy standards. Additionally, the ability of AI to analyze postmarket surveillance data ensures that devices continue to meet safety criteria once deployed in clinical practice.

## Enhancing education and training

The training of cardiovascular interventional providers, particularly fellows and trainees, is another field where AI has the potential to make significant impact. Traditional training methods are limited by time, resources, and opportunities for hands-on learning. AI-driven platforms can, as noted by DeVos et al,^15^ provide immersive, personalized learning experiences that accelerate acquisition of skills allowing the trainees to learn “greater, faster, better” and even add to standardized tests as presented by Nanda et al.^3^^,^^16^ AI-powered extended reality tools allow trainees to practice complex procedures in a risk-free, radiation-free, and contrast-free environment, receiving real-time feedback and performance metrics. Furthermore, Welle et al^17^ explain that AI-powered simulators can adapt to the learner’s skill level, presenting increasingly challenging scenarios to ensure continuous growth. By exposing trainees to a wide range of clinical situations, these tools prepare them for real-world practice in a way that traditional methods cannot.

## The future

The integration of AI into cardiovascular interventions is not complete and not without challenges. Some aspects of our field are only beginning to see the influence of AI, as Holt et al^18^ note regarding congenital heart disease; others are well developed. Regardless of development, all work comes with considerations for ethics, data privacy, and the need for robust validation and standardization that must be addressed. Additionally, the adoption of AI technologies requires significant investment in infrastructure and training for providers; however, the potential benefits far outweigh these challenges. As AI continues to evolve, its applications in cardiovascular interventions will expand, fostering a future where health care is faster, more efficient, and more personalized. The visionary role of AI extends beyond its current capabilities. Imagine a future where AI-powered systems can predict cardiovascular events before they occur, guiding preventive interventions. Picture a world where every patient has access to expert-level care regardless of their location. Envision a health care ecosystem where innovation is accelerated, costs are reduced, and outcomes are consistently optimized.

Collectively, AI represents a transformative force in cardiovascular interventions, addressing critical gaps and unlocking new possibilities. From democratizing expertise and access to revolutionizing device development and enhancing education, AI has the potential to reshape the future of cardiovascular interventions. As we embrace this new era, it is imperative for the medical community to collaborate with cardiovascular industry, regulatory bodies, and policymakers to ensure the responsible and equitable integration of AI. By harnessing its potential, we can advance the field of cardiovascular interventions, ultimately helping our patients to live better and longer lives.
